# Medial malleolus: Operative Or Non-operative (MOON) trial protocol - a prospective randomised controlled trial of operative versus non-operative management of associated medial malleolus fractures in unstable fractures of the ankle

**DOI:** 10.1186/s13063-019-3642-7

**Published:** 2019-09-12

**Authors:** Thomas H. Carter, William M. Oliver, Catriona Graham, Andrew D. Duckworth, Timothy O. White

**Affiliations:** 1 Edinburgh Orthopaedic Trauma, Royal Infirmary of Edinburgh, Edinburgh, EH16 4SA UK; 2 Wellcome Trust Clinical Research Facility, Western General Hospital, Edinburgh, EH4 2XU UK

**Keywords:** Randomised controlled trial, Ankle fractures, Medial malleolus, Fracture fixation, Trauma, Patient outcome

## Abstract

**Background:**

There are limited data reporting the outcome of patients with non-operatively managed medial malleolus fractures compared to those treated surgically in the presence of fibular stabilisation for unstable fractures of the ankle. Conservative management could result in fewer complications, reduced surgical time and lower cost. The purpose of this study is to determine if any difference exists in patient reported and surgical outcomes 1 year after surgery between operative and non-operative treatment of medial malleolar fractures in combination with stabilisation of the lateral malleolus.

**Methods/design:**

This is a single-centre, prospective, randomised controlled trial that aims to randomise 154 participants with an unstable ankle fracture to ‘non-fixation’ (*n* = 77) or ‘fixation’ (n = 77) of an associated well-reduced medial malleolus fracture following fibular stabilisation. The study will include patients ≥ 16 years of age with a closed bimalleolar or trimalleolar ankle fracture who are able to consent, complete questionnaires in the English language, and complete follow-up over a 1-year period. Randomisation will occur intra-operatively when the medial malleolus fracture is deemed ‘well-reduced’, with 2 mm or less of fluoroscopic displacement. The technique for fixation of both the medial and lateral malleoli is at the discretion of the operating surgeon. Patient-reported, observer-rated, and radiographic assessments will be collected at baseline and then at the following post-operative assessment points: 2 weeks, 6 weeks and 1 year. Postal questionnaire outcome data will be collected at 3 and 6 months. The primary outcome measure will be the Olerud Molander Ankle Score (OMAS) at 1 year following surgery. Secondary outcome measures will include the Manchester-Oxford Foot Questionnaire (MOXFQ), EuroQol-5D (EQ-5D), pain, treatment satisfaction, time to return to activity, operative tourniquet time, and complications.

**Discussion:**

There is only one previous randomised trial comparing non-fixation with fixation of associated medial malleolus fractures but that was limited by the lack of baseline patient-reported outcome data and an inferior sample size. This current prospective trial aims to provide high-quality evidence regarding the requirement for medial malleolar fixation in unstable ankle fractures.

**Trial registration:**

ClinicalTrials.gov, NCT03362229. Registered retrospectively on 5 December 2017.

## Background

Ankle fractures are the second most common orthopaedic trauma presentation, accounting for approximately 10% of all fractures presenting at hospital [1]. The annual incidence of ankle fractures is approximately 122–184/100,000 person years (1:800) [2]. According to the Arbeitsgemeinschaft für Osteosynthesefragen (AO) principles, unstable ankle fractures with associated medial malleolar fractures are treated with open reduction and internal fixation (ORIF) [3]. Screw fixation is recommended for the majority of medial fractures, although debate exists regarding the type of screw, the number of screws and the zone of insertion [4–9]. For fractures not amenable to screw fixation due to fragment size, morphology and/or poor bone quality, tension band wiring (TBW) is thought to provide superior fixation [10–12]. Symptomatic metalwork rates following both screw fixation and TBW of medial malleolar fractures are high, prompting development of more novel implants including headless screws, absorbable hardware and knotless systems, with encouraging results [13–18].

A number of retrospective studies have demonstrated that isolated medial malleolus fractures may be treated non-operatively with good patient-reported outcomes and union rates as high as 96% [19, 20]. With an operatively stabilised fibula and a well-reduced medial malleolus fracture, the same principles of non-operative management may be applicable [21]. Only one randomised controlled trial has been conducted on this subject by Hoelsbrekken et al. [22]. The authors recruited 100 patients, with 18 patients (18%) lost to follow-up, leaving 82 patients randomised to a non-operative group (*n* = 45) and an operative group (*n* = 37). There were 51 female (62%) and 31 male (38%) patients. There was no statistically significant difference between the two groups with respect to the Olerud Molander Ankle Score (OMAS) and the American Academy of Foot and Ankle Surgeons (AOFAS) ankle-hindfoot score at a mean follow-up of 44 months. Four cases (8%) of medial malleolar radiographic non-union occurred in the non-operative group, although none of the patients were symptomatic and did not require further treatment. This study was limited by the lack of baseline patient-reported outcome scores and the small sample size.

Building on the current evidence and by including baseline patient-reported outcomes and a larger sample size, the aim of the MOON trial is to determine if any difference exists in the primary outcome measure (OMAS) at the 1-year post-operative stage between non-operative treatment of the medial malleolus in combination with operative fixation of the lateral malleolus and operative fixation of both the medial and lateral malleoli in unstable fractures of the ankle. The secondary aim of this trial is to determine if any difference exists in the complication rate at 1 year post-injury between non-operative treatment of the medial malleolus in combination with operative fixation of the lateral malleolus and operative fixation of both the medial and lateral malleoli.

## Methods

**Fig. 1:**
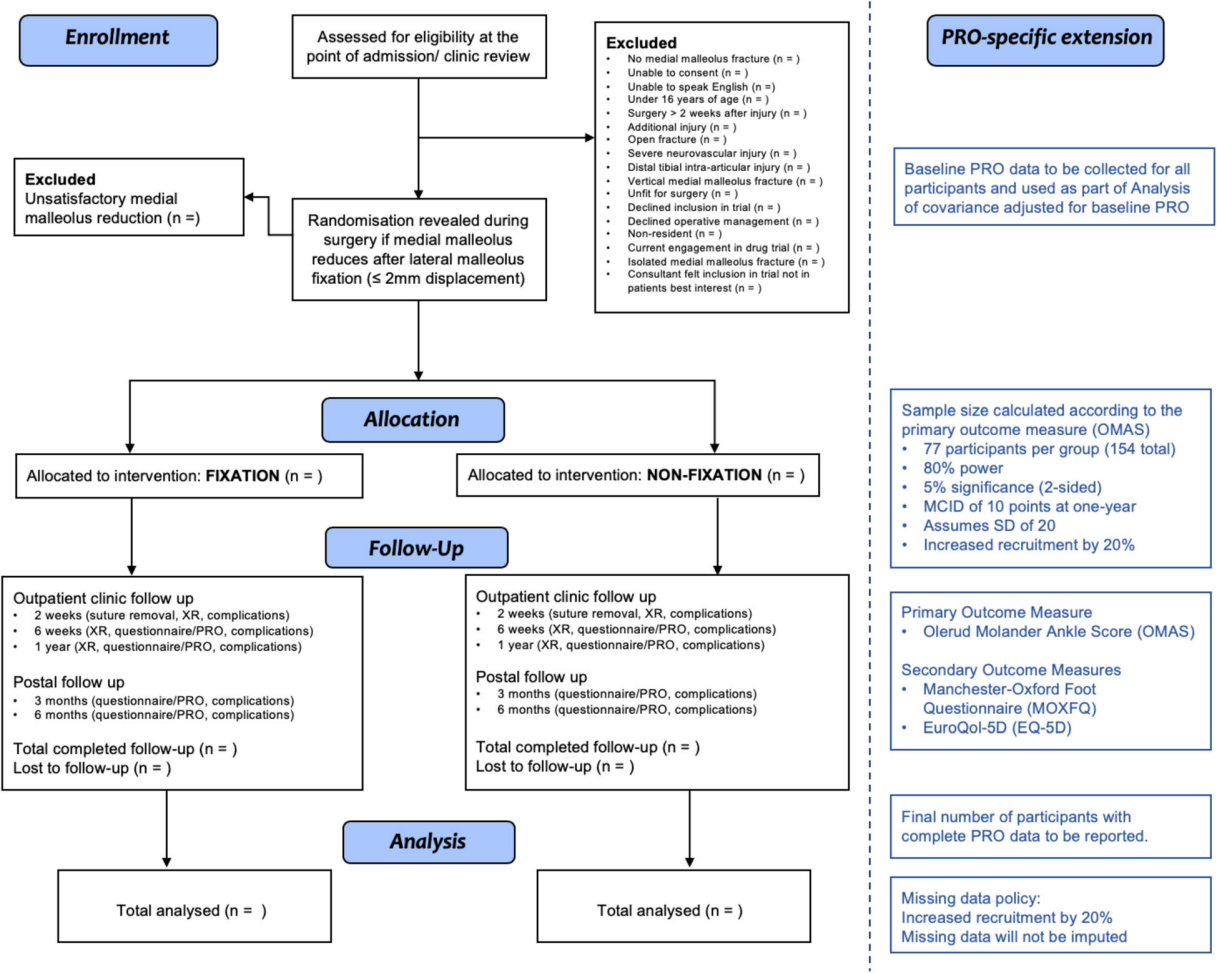
Planned flow of participants. MCID minimal clinically important difference, PRO patient-reported outcome, SD standard deviation, XR x-ray

This single-centre, randomised controlled trial will follow the Consolidated Standards of Reporting Trials (CONSORT) statement [23]. It will be performed in the Edinburgh Orthopaedic Trauma service, Royal Infirmary of Edinburgh, Edinburgh, UK, and has been approved by the South-East Scotland Research Ethics Committee 2 (REC reference 17/SS/0124). The first patient was recruited following satisfactory ethical approval on 24 October 2017. The study was registered with the ClinicalTrials.gov database on 5 December 2017 (ClinicalTrials.gov identifier NCT03362229). This study is co-funded by the Scottish Orthopaedic Research Trust into Trauma (SORT-IT) and an educational grant provided by Acumed (Hillsboro, Oregon, USA). The planned flow of participants is summarised in Fig. 1.

### Inclusion criteria

Inclusion criteria are: 1) age ≥ 16 years; 2) able to consent to treatment; 3) unstable fracture dislocation of the ankle joint requiring operative intervention; 4) closed injury; 5) Weber B and Weber C fractures; and 6) surgery date within 2 weeks of date of fracture.

### Exclusion criteria

Exclusion criteria are: 1) patients unable to comply with post-operative data gathering including completing questionnaires in the English language; 2) additional lower limb injury which may impact on patient rehabilitation; 3) open fracture; 4) confirmed severe associated neurovascular injuries; 5) distal tibial intra-articular fractures/pilon-type injuries; 6) supination-adduction type 2 (SAD-2) fracture configurations with a medial malleolus vertical shear fracture; 7) patients medically unfit for surgery; 8) patients declining operative management; 9) non-residents, unable to return to the unit for follow-up for a period of 1 year; 10) current engagement in a pharmaceutical/drug trial; and 11) where the treating surgeon does not feel that inclusion in the trial is in the patients’ best interest either due to the fracture pattern or patient factors.

### Sample size

The primary outcome measure will be the OMAS. In total, 154 patients (77 per arm) will be required with 80% power and 5% (two-sided) significance to detect a clinically meaningful difference of 10 points on the OMAS scale at 12 months between the two groups [24–26]. This assumes a standard deviation of 20 [27]. The sample size has been increased by 20% to account for any loss to follow-up.

### Randomisation and allocation

Randomisation of treatment will be on a 1:1 ratio. Randomisation will be stratified according to age with a ‘young group’ (< 65 years) and an ‘older group’ (≥ 65 years). Based on retrospective data reporting the epidemiology of ankle fractures at our centre we will stratify on a 3:1 ratio between the ‘young’ and ‘old’. This will be factored into the computer-generated randomisation schedule, which will utilise a block design of mixed block sizes and will be generated by an independent statistician employed through the local university research methodology department. A member of staff independent of the trial will use this list to create 154 opaque sequentially sealed envelopes clearly distinguishing those for the young and old groups separately. Contained within each envelope will be a sticker bearing the words ‘Fixation’ or ‘Non-fixation’, which will be placed onto the study consent form.

**Fig. 2:**
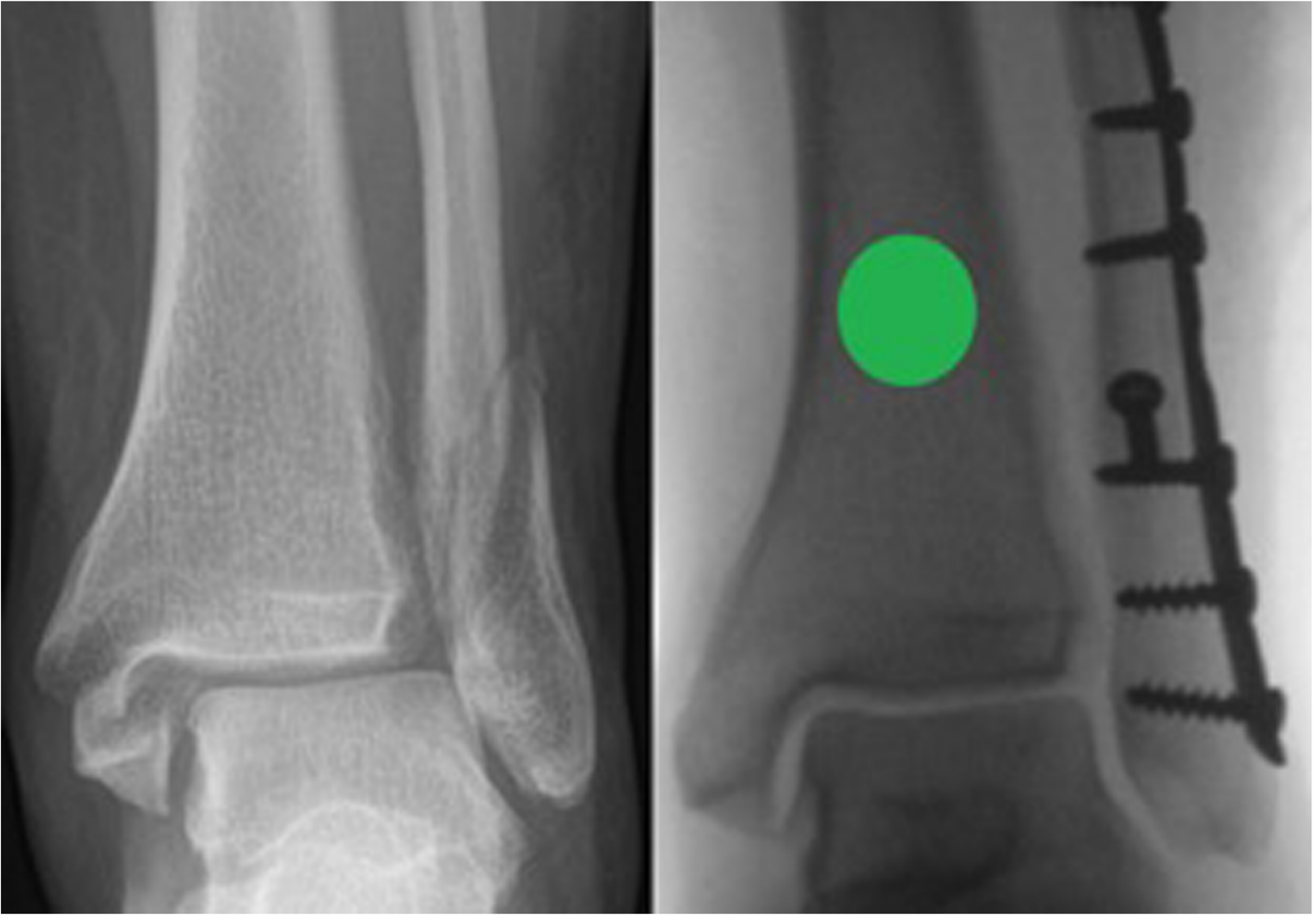
Anteroposterior radiograph of a supination-external rotation stage IV fracture at the time of injury and following fibular stabilisation, demonstrating an anatomically reduced medial malleolus. This patient would be eligible for intra-operative randomisation

**Fig. 3:**
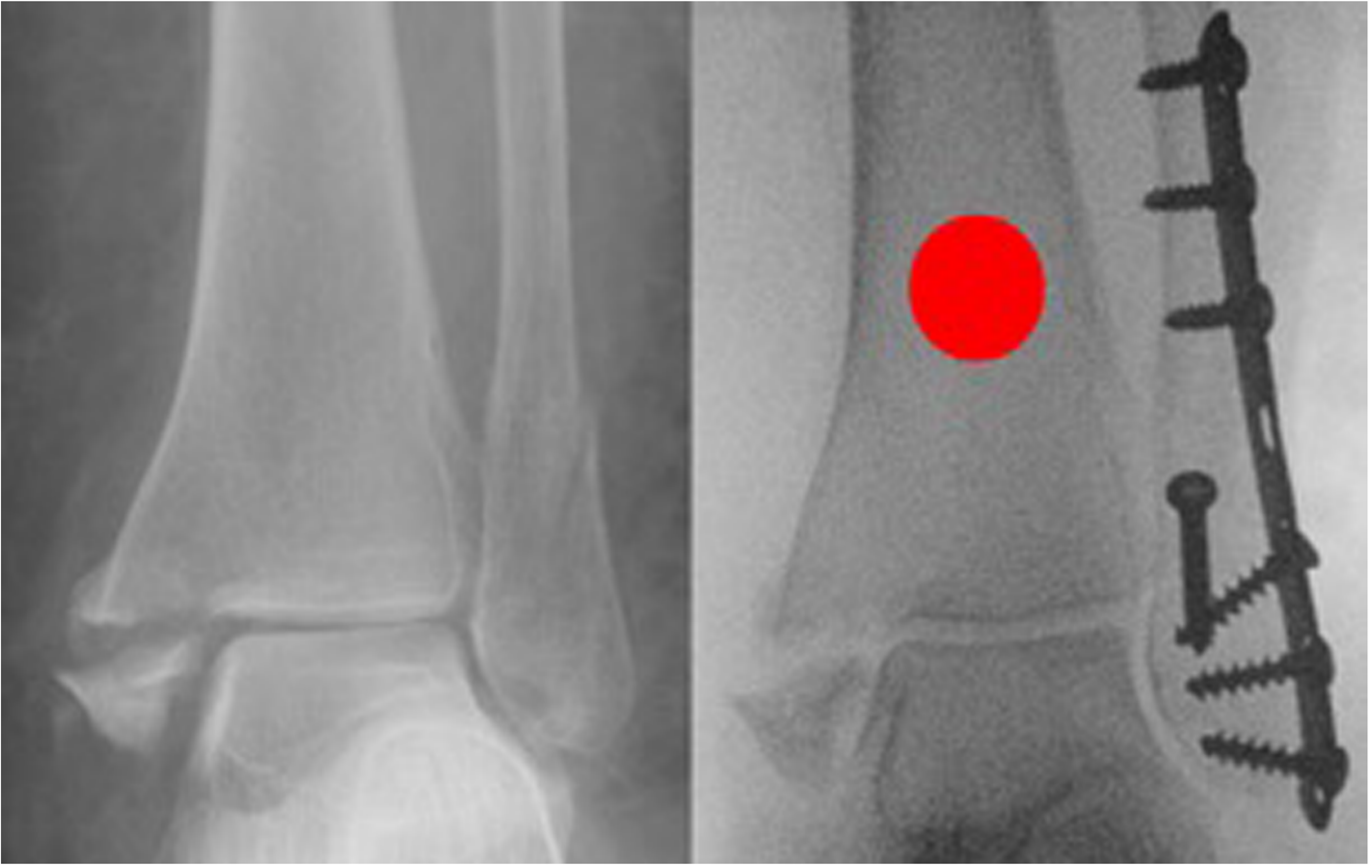
Anteroposterior radiograph of a supination-external rotation stage IV fracture at the time of injury and following fibular stabilisation. The medial malleolus has not reduced to acceptable limits, likely incarcerated by soft tissue. This patient would not be eligible for intra-operative randomisation

Participants will be identified by members of the on-call admitting team as they attend the local hospital emergency department (ED) or who are referred to the outpatient trauma clinic. The trial will be introduced by a member of staff and a patient information sheet (PIS) specific to the trial will be given to the patient to consider. Once the patient has had time to read and consider the information, they will be approached by a member of the research team (one of four) who will clarify any points of uncertainty. The patient will be asked to sign a consent form providing their permission to enter the study and the next available randomisation envelope (age-dependent) will accompany the consent form into the operating theatre with the patient. The participant will not be aware of the result of randomisation at the point of study enrolment. The envelope will remain unopened until the operating surgeon has confirmed that the reduction of the medial malleolus fracture on an anteroposterior view is acceptable following fibular stabilisation. The envelope will be opened by a member of theatre staff who is independent of the trial. In the event that the fracture is not well-reduced, the envelope is returned in sequence back to the study office and the patient is withdrawn from the study. In the case of a trimalleolar fracture with an associated posterior malleolar fracture, fracture fixation will be at the discretion of the operating surgeon and influenced by the size of the fragment, articular congruity and presence of posterior talar subluxation. Examples of an acceptable and unacceptable intra-operative medial malleolar reduction are shown in Figs. 2 and 3.

### Interventions

#### Non-fixation

Participants randomised to non-fixation of their well-reduced medial malleolus fracture will not require further intervention on the medial side. Wound closure on the lateral side, post-operative immobilisation and weight bearing restriction will be at the discretion of the operating surgeon. As a general rule in our service we aim to fit all patients with a removable orthosis and allow them to immediately fully weight bear wherever possible, only restricting in the case of a syndesmotic injury or peripheral neuropathy for between 6 and 8 weeks. The presence or absence of medial malleolus fixation should not influence the surgeon’s decision with respect to the post-operative management.

#### Fixation

Participants randomised to fixation of their well-reduced medial malleolus fracture will be treated with standard medial malleolar fixation techniques at the discretion of the operating surgeon, the most common of which involves one or two 3.5-mm partially threaded cancellous lag screw (35–45 mm length) inserted at 90^o^ to the fracture, following a satisfactory open reduction. Other techniques may include tension band wire construct and Kirschner wires, appropriate for size and integrity of the malleolar fragment. Wound closure and post-operative management are at the discretion of the operating surgeon as in the non-fixation trial arm.

### Blinding

Given the invasive nature of surgery those patients randomised to ‘Fixation’ will have an additional surgical wound on the medial aspect of their ankle and those who are randomised to ‘Non-fixation’ will not. It is therefore not possible to blind either the surgical team or the patients in this trial.

### Outcome assessment

**Fig. 4:**
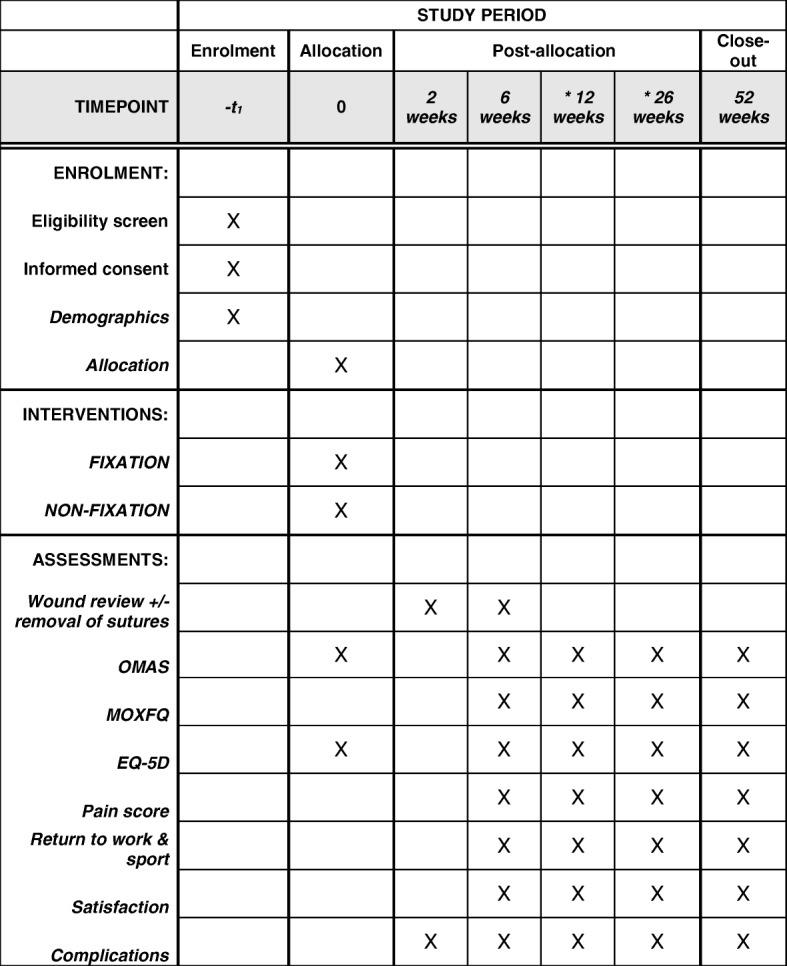
Schedule of enrolment, interventions, and assessments. *Data collected through postal questionnaire. EQ-5D EuroQol 5D, MOXFQ Manchester-Oxford Foot Questionnaire, OMAS Olerud Molander Ankle Score

All participants, regardless of randomisation, will be reviewed back in the research outpatient clinic at 2 weeks, 6 weeks and at 1 year post-intervention. During the 2-week post-operative assessment, the wounds will be inspected, sutures removed by a dressings nurse and the patient will undergo anteroposterior (AP) and lateral radiographs of the ankle joint to ensure satisfactory talar reduction and no displacement of the metalwork or fractures. Immediate complications including infection, wound dehiscence and venous thromboembolic (VTE) disease will be documented. The 6-week, 8-week and 1-year assessment points will include further weightbearing AP and lateral radiographs to assess fracture union, talar reduction and radiographic evidence of osteoarthritis. Patients will complete the patient-reported outcome measures of OMAS, Manchester-Oxford Foot Questionnaire (MOXFQ), EuroQol 5D (EQ-5D), pain and treatment satisfaction on visual analogue scales (VAS), and return to work and sport. Complications will be documented. Physiotherapy will be organised at the discretion of the clinician reviewing the patient in the clinic to begin following the 6-week assessment. Postal questionnaires will be sent out at 3 and 6 months after surgery collecting the same outcome scores. The schedule of enrolment, interventions, and assessments as per the Standard Protocol Items: Recommendations for Interventional Trials (SPIRIT) is summarised in Fig. 4.

### Primary outcome

The primary outcome measure will be the OMAS at 1 year following surgery [28]. This is a validated lower limb outcome score that has been used in large Health Technology Assessment (HTA)-funded trials concerning the ankle [24, 27]. This scoring system assesses patient-reported outcome across nine parameters: pain, stiffness, swelling, stair climbing, running, jumping, squatting, supports, and work/activities of daily living. A final score is calculated from 0 to 100, with 100 representing a better functional outcome. A recent study found that the OMAS had acceptable levels of internal consistency and test-retest validity, and correlated strongly with other lower limb injury outcome scoring systems and measures of general health, including the EQ-5D [29]. The primary null hypothesis is that there is no difference in outcome (primary outcome measure, OMAS) after 1 year between fixation of associated medial malleolus fractures and non-fixation in patients undergoing surgery for an unstable fracture of the ankle.

### Secondary outcomes

Secondary outcome measures include: 1) MOXFQ (Isis Innovation Ltd., Oxford, UK) [30], a validated and reliable patient-reported outcome measure for surgery of the foot and ankle, consisting of 16 items from three domains: walking/standing (seven items), pain (five items) and social interaction (four items) where each item is scored on a five-point Likert scale ranging from no limitation to maximum limitation (0, 1, 2, 3, 4); a raw score out of 64 is then converted to a 0–100 metric score, with 100 representing a better functional outcome; 2) EQ-5D [31], a standardised instrument for use as a measure of health outcome; 3) pain assessment measured on a VAS from 0 to 100 with 100 indicating no pain; 4) treatment satisfaction measured on a VAS from 0 to 100 with 100 indicating extremely satisfied; 5) time taken to return to work and sport (if applicable); 6) operative tourniquet time; and 7) complications including superficial and deep infection, nerve injury, chronic pain, failure of fixation, non-union, metalwork complications and re-operation.

### Statistical analysis

The analysis of primary outcome data will include participants who have completed their 12-month follow-up, and will be analysed on an intention-to-treat basis. Statistical analysis will be performed by a senior statistician who is independent of the study and employed through the local university research methodology department. The primary outcome measure (OMAS at 1 year) will be compared between the two treatment groups using a two-way sample *t* test or non-parametric equivalent, dictated by the normality of the data. An analysis of covariance adjusting for age and the baseline patient-reported outcome measure (OMAS) will be performed. This method will also be used to compare other continuous outcome measure scores. The OMAS will also be collected at multiple time points to calculate change in outcome over time. This will be determined by fitting a linear regression to the 6-week to 12-month values of each participant and comparing the slope of the regression line between treatment arms. Comparison of binary outcomes such as the presence of non-union, infection and re-operation will be compared between the two treatment groups using a binominal test for comparison of proportions. Two-tailed *p* values will be presented wherever possible and a *p* value < 0.05 will be used to indicate statistical significance.

### Missing data

The study sample size has been increased by 20% to account for loss to follow-up at the primary outcome point (12 months). The primary outcome point will be collected at the final outpatient clinic appointment and, as such, it is anticipated that missing data for the primary outcome will be low. The participant dropout rate for each arm of the trial will be reported and compared. If the participant dropout rate at the primary outcome point is high, a sensitivity analysis may be performed. Missing data at the intermediate data collection points are most likely to occur at the 3- and 6-month assessment points as data collection is via postal questionnaire. However, by performing an analysis on change over time through fitting regression models the impact of missing data will be minimised.

### Patient safety

Secondary outcome measures include complications, which will be closely monitored in both arms of the trial. This trial will be conducted in line with the Academic and Clinical Central Office for Research and Development (ACCORD) published guidelines: Identifying, Recording and Reporting Adverse Events for Clinical Investigations of Medical Devices.

## Discussion

The most basic fracture classification system, devised by Pervical Pott, describes the number of malleoli involved—unimalleolar, bimalleolar and trimalleolar [32]. The lateral and medial malleoli are important contributors to ankle stability in conjunction with their associated ligaments—the lateral ligament complex and medial/deltoid ligament, respectively.

There has been considerable historical debate regarding the significance of the contribution of the medial malleolus to ankle joint stability. Yablon et al. concluded that the lateral malleolus was fundamental in anatomical reduction of bimalleolar fracture patterns with the talus ‘always faithfully followed the lateral malleolus upon reduction’ [33]. This was confirmed with cadaveric ankle stress testing which found that ankle stability was minimally affected upon sectioning of the deltoid ligament or fracture of the medial malleolus. Yablon’s theory went against the views of others including Hughes who hypothesised it was in fact the medial malleolus which helped to re-establish a stable and congruent mortise [34]. The majority of surgeons therefore considered the medial malleolus to be the most important stabiliser and consequently unstable bimalleolar ankle fracture dislocations were commonly treated with open reduction and internal fixation of the medial malleolus in conjunction with closed reduction of the lateral malleolus.

When assessing load bearing in the ankle joint, the majority of the bodyweight is distributed over the central zone of the distal tibial plafond. During standing and walking, 90% of the loading occurs in this area with the remaining load being shared between the medial and lateral malleoli [35]. Consequently, good results have been published in patient cohorts with conservatively managed isolated medial malleolus fractures [19, 20]. Herscovici et al. identified 57 patients with conservatively managed isolated medial malleolus fractures, accepting any degree of fracture reduction [19]. Only two cases required further intervention with an overall union rate of 96%. Patients reported good outcomes as per the Short Form-36 (SF-36) and AOFAS ankle-hindfoot score. Importantly there were no cases of medial instability, skin compromise, malalignment of the mortise or post-traumatic degenerative changes after a mean 3-year follow-up. They concluded that isolated medial malleolus fractures could be treated non-operatively, but consideration should be given to fixation in the cases of bimalleolar and trimalleolar fracture dislocations, which were deemed more inherently unstable. More recently Hanhisuanto et al. concluded that medial malleolar reduction was critical for a good patient outcome when managed conservatively, defining 2 mm or less of displacement as the absolute acceptable cut-off [20].

Any operation, especially on the foot and ankle, is associated with a risk of surgical site infection (SSI), particularly in elderly patients who may have contributing risk factors such as diabetes, immunosuppression and peripheral vascular disease. Infection rates between 8% and 13% have been quoted, with up to 10% requiring further surgery for removal of metalwork or wound debridement [36, 37]. With this in mind, the benefits of minimising skin incisions and implantation of metalwork are clear.

Following a review of the recent literature, this randomised controlled trial will provide superior statistical power when assessing the impact of medial malleolus fixation on patient-reported and surgical outcomes following bimalleolar or trimalleolar fractures. Given the varied practice in ankle fracture surgery including implant selection for fibular stabilisation, medial malleolar fracture fixation, wound closure, post-operative immobilisation and weight-bearing restrictions, the study has a pragmatic design to reproduce day-to-day trauma care [38, 39].

One anticipated difficulty will be intra-operative randomisation. We have defined an acceptable reduction as no more than 2 mm of displacement as measured fluoroscopically on an AP view, based on the recent evidence [20, 22]. During surgery there will be subjective variability in the surgeon’s accurate determination of displacement with the fluoroscopy equipment available. Therefore, the surgeon will have to decide whether the displacement is acceptable by assessing the overall shape and alignment of the mortise. Revealing the allocation of randomisation before the reduction has been assessed would create potential bias regarding the interpretation of reduction quality. It is crucial that the envelope is not opened until authorised to do so by the operating surgeon and this must be performed by an independent member of staff. A research co-ordinator will be present in theatre to ensure the result of randomisation is not revealed until the correct stage in the surgical procedure. If the patient is no longer eligible the envelope must be returned immediately to the study office in order to prevent disruption to the randomisation sequence.

A limitation of the study relates to the single-centre design. However, the study centre provides orthopaedic care to a population of approximately 850,000 and has 12 orthopaedic trauma consultants who collectively provide operative intervention for approximately 450 ankle fractures per year. The authors feel the results will therefore be reliably extrapolated to the wider orthopaedic community. Patient-reported outcome scores are being increasingly used in orthopaedic trials with clear benefits—the focus of outcome is centred on the patient, and outcome scores can be collected remotely. The OMAS (primary outcome) may be limited as it focuses more on physical symptoms (pain, stiffness and swelling) and patient performance (running, jumping, squatting), whilst neglecting the impact on emotional/mental well-being. It is therefore important to collect data on a general health outcome (EQ-5D) as part of the assessment in this prospective trial.

### Trial status

The first patient was recruited on 24 October 2017. The expected date of completion is February 2021.

## Data Availability

Not applicable.

## Abbreviations

ACCORD: Academic and Clinical Central Office for Research and Development
AO: Arbeitsgemeinschaft für Osteosynthesefragen
AOFAS: American Academy of Foot and Ankle Surgeons
AP: Anteroposterior
CONSORT: Consolidated Standards of Reporting Trials
ED: Emergency department
EQ-5D: EuroQol 5D
HTA: Health Technology Assessment
MOXFQ: Manchester-Oxford Foot Questionnaire
OMAS: Olerud Molander Ankle Score
ORIF: Open reduction and internal fixation
PIS: Patient information sheet
REC: Research Ethics Committee
SAD: Supination adduction
SF-36: Short Form-36
SORT-IT: Scottish Orthopaedic Trust into Trauma
SPIRIT: Standard Protocol Items: Recommendations for Interventional Trials
SSI: Surgical site infection
TBW: Tension band wiring
VAS: Visual analogue scale
VTE: Venous thromboembolic
